# Classifying Variants of Undetermined Significance in BRCA2 with Protein Likelihood Ratios

**DOI:** 10.4137/cin.s618

**Published:** 2008-04-18

**Authors:** Rachel Karchin, Mukesh Agarwal, Andrej Sali, Fergus Couch, Mary S. Beattie

**Affiliations:** 1 Department of Biomedical Engineering, Institute for Computational Medicine, Johns Hopkins University; 2 Mayo Clinic College of Medicine, Department of Laboratory Medicine and Pathology; 3 Departments of Biopharmaceutical Sciences and Pharmaceutical Chemistry, and California Institute for Quantitative Biomedical Research, University of California San Francisco; 4 Familial Cancer Risk Core Facility and Cancer Risk Program, University of California San Francisco; 5 Department of Medicine, University of California San Francisco

**Keywords:** breast cancer, risk assessment, mutagenesis, cancer susceptibility genes, bioinformatics and computational biology, missense variants

## Abstract

**Background:**

Missense (aminoacid changing) variants found in cancer predisposition genes often create difficulties when clinically interpreting genetic testing results. Although bioinformatics has developed approaches to predicting the impact of these variants, many of these approaches have not been readily applicable in the clinical setting. Bioinformatics approaches for predicting the impact of these variants have not yet found their footing in clinical practice because 1) interpreting the medical relevance of predictive scores is difficult; 2) the relationship between bioinformatics “predictors” (sequence conservation, protein structure) and cancer susceptibility is not understood.

**Methodology/Principal Findings:**

We present a computational method that produces a probabilistic likelihood ratio predictive of whether a missense variant impairs protein function. We apply the method to a tumor suppressor gene, BRCA2, whose loss of function is important to cancer susceptibility. Protein likelihood ratios are computed for 229 unclassified variants found in individuals from high-risk breast/ovarian cancer families. We map the variants onto a protein structure model, and suggest that a cluster of predicted deleterious variants in the BRCA2 OB1 domain may destabilize BRCA2 and a protein binding partner, the small acidic protein DSS1. We compare our predictions with variant “re-classifications” provided by Myriad Genetics, a biotechnology company that holds the patent on BRCA2 genetic testing in the U.S., and with classifications made by an established medical genetics model [1]. Our approach uses bioinformatics data that is independent of these genetics-based classifications and yet shows significant agreement with them. Preliminary results indicate that our method is less likely to make false positive errors than other bioinformatics methods, which were designed to predict the impact of missense mutations in general.

**Conclusions/Significance:**

Missense mutations are the most common disease-producing genetic variants. We present a fast, scalable bioinformatics method that integrates information about protein sequence, conservation, and structure in a likelihood ratio that can be integrated with medical genetics likelihood ratios. The protein likelihood ratio, together with medical genetics likelihood ratios, can be used by clinicians and counselors to communicate the relevance of a VUS to the individual who has that VUS. The approach described here is generalizable to regions of any tumor suppressor gene that have been structurally determined by X-ray crystallography or for which a protein homology model can be built.

## Introduction

The promise of “personalized medicine” using genetic testing to assist with estimation of disease risk, brings with it the reality of receiving test results that are difficult to interpret. Many sequence variants in cancer predisposition genes are of uncertain clinical significance. This creates a clinical problem for individuals desiring individualized risk assessment and for providers recommending risk reducing strategies [2, 3]. Missense mutations, which arise from a single DNA base substitution and cause an amino acid substitution in the ensuing protein, represent the most common of all known disease producing genetic variants (Human Gene Mutation Database, http://www.hgmd.cf.ac.uk/ac/index.php). With the current availability of comprehensive genetic testing for many genes, and with the future prospect of sequencing whole genomes in individuals, it is essential to understand how missense mutations affect protein function and whether individual missense mutations predispose to disease.

For most comprehensive genetic tests, there are three possible results: positive (known disease-associated mutation), negative (no known disease-associated mutation found) and Variant of Undetermined Significance (*VUS*: sequence variant found, but association with disease is unclear). In the case of genetic testing for susceptibility to hereditary breast and ovarian cancer, considerable differences in risk [4–6] and very different approaches to prevention [7] would result if a VUS is presumed to be deleterious or neutral. Considering the population presenting for BRCA testing in the U.S., the chance of receiving VUS results is approximately 5% in Caucasians and up to 30% in non-Caucasians [2].

In 2004, Goldgar et al. developed an integrated model [8] to evaluate VUS, combining epidemiological observations with data from sequence analysis. This model combines several independent likelihood ratios that model familial segregation, co-occurrence of a VUS with a known deleterious mutation, sequence conservation and aminoacid change severity. Taken together, the likelihood ratios provide an estimate of the *odds of causality* for a single VUS. An odds in favor of causality of over 1000:1 is considered pathogenic and an odds against causality of more than 100:1 is considered neutral. Subsequent research has incorporated a modified Grantham analysis component into the model [9], and has also incorporated histopathological and immunohistochemical (IHC) data as well as loss of heterozygosity studies [2].

For families at high risk of hereditary breast and ovarian cancer, standard of care practices recommend genetic testing for mutations in BRCA1 and BRCA2 (National Comprehensive Cancer Network. Clinical Practice Guidelines in Oncology, Genetic/familial high risk assessment: breast and ovarian cancer http://www.nccn.org/professionals/physician_gls). Of these two genes, BRCA2 is larger, and recent data suggests that BRCA2 mutations, on a population level, may be more prevalent than BRCA1 mutations [10]. The spectrum of cancers associated with BRCA2 mutations likely includes other cancers as well, such as pancreatic, prostate, stomach, melanoma, gallbladder, and bile duct cancers [11, 12].

We have previously shown that supervised learning algorithms, developed in the computational machine learning community, can be useful in predicting when a VUS in the breast cancer C-terminal (BRCT) domains of BRCA1 causes impaired protein function, and that such predictions are consistent with available genetic information for selected VUS [13]. These algorithms are capable of integrating multiple properties predictive of how a VUS will impact protein structure and function, including sequence conservation, aminoacid change severity and importantly, impact on the local protein structure environment. This method used a consensus vote of three different supervised learning algorithms to make predictions. However the output was not a likelihood ratio that could be included in estimates of overall causality odds, the quantity of interest from a clinical standpoint.

Here we have developed a method of transforming the output of one supervised learning algorithm, a support vector machine [15], into a likelihood ratio that can be combined with other independent predictors to aid the classification of previously undetermined variants in BRCA2. Currently we do not have a gold standard for BRCA VUS prediction, which makes it difficult to evaluate the performance of prediction models. A gold standard carefully classifies data, has face validity from its users, and has literature as well as the “test of time” to support its utility. We suggest that in a field where there is not yet a gold standard, it is useful to look for consensus between predictors that use information from independent sources. Thus we compare our method to other computational biology methods, according to their consensus with two models based on medical genetics (Table 1, Table 2).

## Methods

### Supervised learning

**Figure 1:**
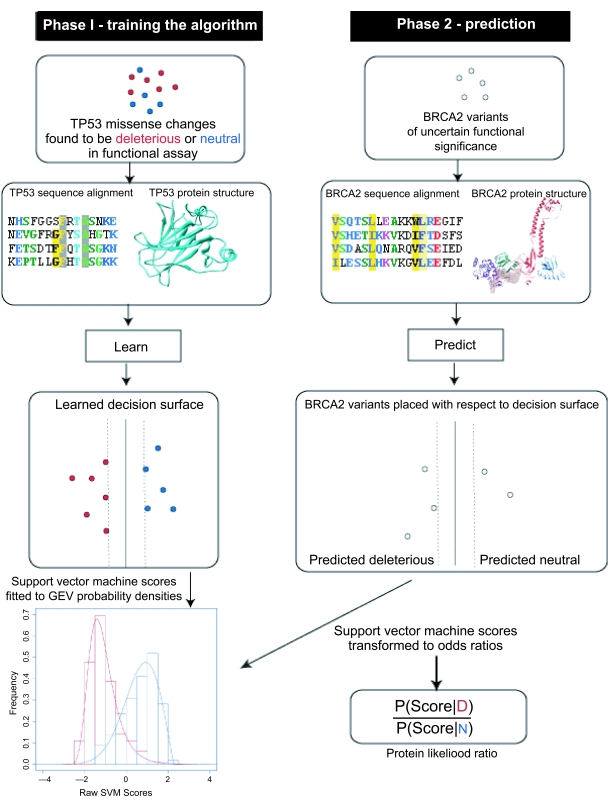
**Protein likelihood ratio algorithm.** In the first phase, a support vector machine learns a decision surface that separates deleterious (red) and (blue) neutral missense changes in TP53, based on predictive properties from protein sequence and structure. The discriminant scores of TP53 missense changes are fit to a mixture of Generalized Extreme Value (GEV) probability densities (Red = deleterious scores, Blue = neutral scores). In the second phase, a BRCA2 missense variant is classified as deleterious or neutral using equivalent predictive properties. The discriminant score is transformed into a likelihood ratio that quantifies the odds that the score reflects a deleterious (D) or neutral (N) missense variant.

The protein likelihood scores are built “on top of ” a support vector machine supervised learning algorithm [15–17]. We first trained a support vector machine to predict whether missense variants in the BRCA2 DNA-binding domains are deleterious or neutral (*e1071* R package [18] radial basis kernel with parameters g = 0.0625, c = 1.0) . There are two phases to support vector machine learning and prediction (Fig. 1). In the first phase, the algorithm is shown a training set of missense changes whose class is known and learns a separating decision “surface”. As described previously [19], each missense change is represented by 16 predictive properties that describe sequence conservation of the position where the amino acid substitution occurs, the residue solvent accessibility, geometry of the protein backbone at the position, the amount of strain induced on protein conformation by the substitution, and physiochemical properties of amino acid residues. In the second phase, the decision surface is used to predict whether missense changes whose class is not known are deleterious or neutral, and each prediction is transformed into a protein likelihood ratio.

### Training set

We use a collection of missense changes engineered in a structure-function study of TP53 as a training set. The study compared the transactivation activity of 2314 TP53 missense mutants to wild-type [20]. Mutant constructs were tested for trans-activation of a reporter gene downstream of eight known TP53 transcriptional enhancer sites. Data was downloaded from the IARC TP53 website (http://www-p53.iarc.fr) and we identified 618 “extreme phenotype” missense mutants that were incapable (398 total) or capable (220 total) of activating transcription for all eight of the transcriptional enhancers tested in the assay. These 618 mutants are in the core DNA-binding domain of TP53 where 97% of both germline and somatic missense changes have been observed (http://www-p53.iarc.fr). Because the nonfunctional mutants predominate in this training set, we use “class weights” when training the support vector machine to avoid building a model that overpredicts deleterious mutations. Class weights (*w_D_* for non-functional mutants *,w_N_* for functional mutants) were set to *w_D_* = 1.3 and *w_N_* = 1.7 to downweight the majority class and upweight the minority class, according to the proportions that they are found in the training set and to ensure that the original sum of class weights was unchanged (in the “unweighted” case, each class has a default weight of 1.0), so that *w_D_* + *w_N_* = *2 and 0.35 w_D_* = 0.65 *w_N_*

### Testing set

We downloaded all missense variants in the C-terminal DNA-binding domains of BRCA2 collected in the Breast Information Core (BIC) database (http://research.nhgri.nih.gov/bic/, 31-Jan-2007 update). The C-terminal domains of the human BRCA2 protein (exons 15–25, codons 2479–3191) are of particular interest with respect to cancer susceptibility because: 1) they are the most evolutionarily conserved region of the protein [21], 2) they are important for the role of BRCA2 in DNA repair and homologous recombination [21, 22]; 3) *in vitro* assays have shown that this region binds molecules critical for BRCA2 function (single-stranded DNA and the protein DSS1) [21, 23] and 4) although there is no high-quality X-ray crystal structure of the human BRCA2 C-terminal DNA-binding domains, it is possible to build a good quality protein structure model from X-ray crystal structures of these domains from two species closely related to human (rat and mouse) [21].

Machine learning requires that predictive features are calculated for all examples in the training set and also for all examples whose class we want to predict. Thus, we require protein structures and multiple sequence alignments for both the core DNA-binding domain of TP53 and the C-terminal domains of BRCA2. We now describe how the protein structure coordinates and multiple sequence alignments for TP53 and BRCA2 were obtained.

### Protein structures

**Figure 2:**
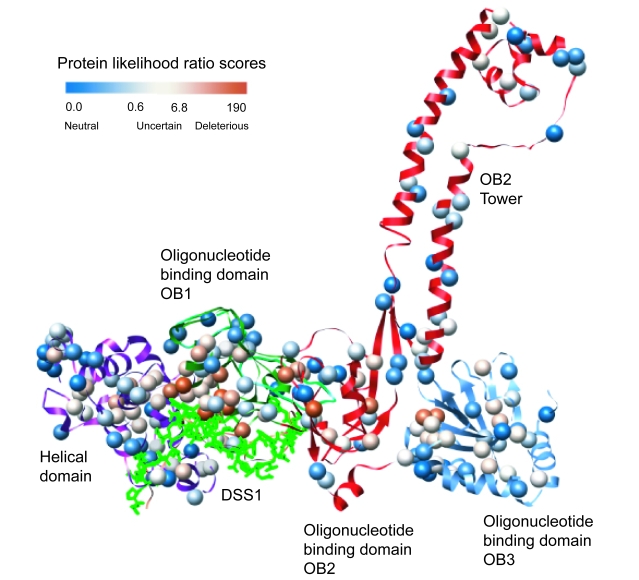
**Homology model of human BRCA2 C-terminal DNA binding domains with positions of Variants of Undetermined Significance (VUS) in the Breast Information Core database (BIC) (http://research.nhgri.nih.gov/bic/ January 31, 2007 update)**. The VUS are colored by their protein likelihood ratio scores with lowest (predicted neutral) in blue, uncertain in white, and highest (predicted deleterious) in red. The OB1 domain shows enrichment of predicted deleterious near its binding site of the small acidic protein DSS1.

We downloaded an X-ray crystal structure of the TP53 core DNA-binding domain from the Protein Data Bank [24] (1kzy chain A in complex with the 53BP1 BRCT domains [25]). Currently there is no X-ray crystal or NMR tertiary structure data for the human BRCA2 protein. Therefore, we built a homology model of human BRCA2, in complex with the small acidic protein DSS1, using MODELLER 9.1 [26] (Fig. 2). We employed three hand-aligned mouse and rat structures of the BRCA2-DSS1 complex as templates (PDB codes 1mje, 1miu, 1iyj [21]), built an ensemble of 50 models and selected the model with best MODELLER objective function. This model was examined for poor quality regions with the DOPE statistical potential [27] and two loops were subjected to further refinement with MODELLER’s loop modeling routines. For both TP53 and BRCA2, each missense change was simulated using MODELLER’s mutate_model protocol as described previously [19]. Model coordinates are available on request.

### Protein sequence alignments

Protein sequences of human TP53 (P04637) and BRCA2 (P51587) were downloaded from UNI-PROT [28]. We extracted the sequence of the TP53 core DNA-binding domain and the BRCA2 DNA-binding domains by hand. The domain-specific sequences were used as input to the SAM-T2K iterative multiple sequence alignment-building algorithm [29]. We use the multiple sequence alignments to compute two predictive properties that quantify the evolutionary importance of each amino acid residue position where a missense mutant occurs, as described previously [19]. The TP53 and BRCA2 alignments are available upon request.

### Support vector machine predictions

The support vector machine uses the training set and predictive features to learn a “decision surface” that separates deleterious (or loss of function) amino acid changes from those that are biologically neutral (Fig. 1). In general, this learning algorithm finds a unique decision surface, which maximally separates the two classes. In the second phase, the decision surface is used to assign a *discriminant* score to each BRCA2 VUS. Discriminant scores less than zero predict that the VUS will induce loss of BRCA2 function. However, these scores are not in a form we can directly incorporate into an odds-of-causality ratio.

### Protein likelihood ratios

**Figure 3:**
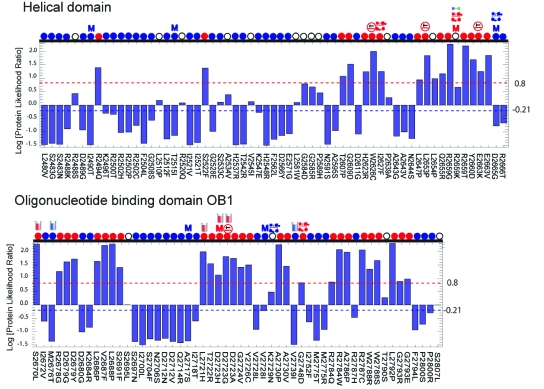

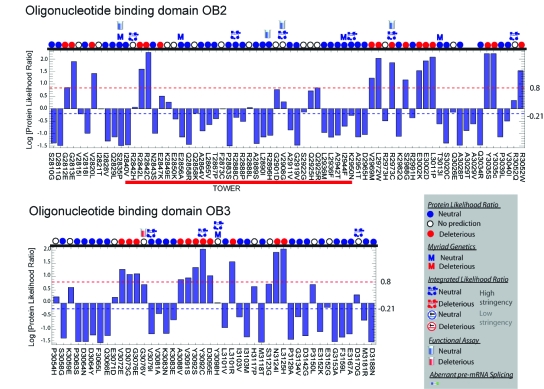
Figure 3a, 3b. Protein Likelihood Ratios for 223 BIC VUS in the C-terminal DNA binding domains of BRCA2. Protein likelihood ratios are shown on a Log_10_ scale with classifications of Deleterious, Neutral, or Not Predicted. Variants are classified as Neutral when Protein Likelihood Ratio <= 0.61 (blue dotted line at −0.21 Log scale) and Deleterious when Protein Likelihood Ratio >= 6.8 (red dotted line at 0.8 Log scale). Icons shown above each variant indicate the Protein Likelihood Ratio classification (red, blue, and white circles), Myriad Genetics classification (red and blue M’s), functional data from a Homology Directed Repair Assay [42] (Supplementary Table 2, red and blue test tubes), and the Integrated Likelihood model [1, 8] High Stringency (Deleterious classification requires odds of 1000:1) and Low Stringency (Deleterious classification requires odds of 100:1).

To incorporate our method into the combined odds of causality model that has gained much acceptance in the genetic epidemiology community [1,8] requires the likelihood ratio P(*S* | *D*)/P(*S* | *N*) for each variant of interest, where S is the discriminant score. Standard machine learning methods can yield posterior probabilities of the form P(*D* | *S*) and P(*N* | *S*) and thus posterior likelihood ratios P(*D* | *S*)/P(*N* | *S*). If the prior probability that a variant is deleterious or neutral were known, we could infer this likelihood ratio from the posterior, using Bayes’ Rule. However, these priors are not currently known. Here we use an alternative method to transform discriminant scores into our desired likelihood ratios. We first express the distribution of discriminant scores for deleterious TP53 missense changes as a parameterized probability distribution of known functional form *P*(*S* | *D*, θ*_D_*) that quantifies the probability of seeing a particular discriminant score *S* when the mutant induces loss of function. Likewise, we express the distribution of neutral scores in a known functional form *P*(*S*|*D*, θ*_N_*). The protein likelihood ratio is then calculated as *P*(*S*|*D*, θ*_D_*)/*P*(*S*|*N*, θ*_N_*), yielding an odds ratio in favor of loss of function. Histograms of “deleterious” and “neutral” TP53 discriminant scores (Fig. 1) suggest that the scores are distributed as Generalized Extreme Value (GEV) distributions. We use maximum likelihood to fit GEV parameters for deleterious and neutral mutants using the *ismev* R package [18]. This approach yields GEV parameters for deleterious mutants (θ_D_) −1.5 (location), 0.66 (scale), 0.015 (shape) and GEV parameters for neutral mutants (θ*_N_*) 0.7 (location), 0.78 (scale), − 0.51 (shape). We assign thresholds for prediction confidence based on available data from medical genetics studies. Confident predictions are those whose likelihood ratios are either 1) larger than the variant with the smallest likelihood ratio but greater than 1.0 that has been reclassified as “Deleterious” or “Suspected Deleterious” by Myriad Genetics or been shown to have an Integrated Likelihood Ratio > 1,000; or 2) smaller than the ratio of the variant with the largest likelihood but less than 1.0 that has been reclassified as neutral or “Polymorphism” by Myriad (Fig. 3a, 3b, Supplementary Table 1). Predictions for VUS that lie between the thresholds are not considered reliable. These thresholds can be modified as new information becomes available.

### Model fit

We measured model goodness of fit for our parameterizations of *P*(*S* | *D*, θ*_D_*) and *P*(*S* | *N*, θ*_N_*) with Fisher’s Exact test (two-sided). There was no significant difference between the score distributions and their expected frequencies indicating that the parameterizations are a good fit to the scores. (for *P*(*S* | *D*, θ*_D_*) n = 398, P = 0.8122, for *P*(*S* | *N*, θ*_N_*) n = 220, P = 0.7647, alpha = 0.05 for both tests).

### Validation set

We evaluated the Protein Likelihood Ratios with a validation set consisting of ten variants that have Myriad Genetics reclassifications (all those available to us in the C-terminal domains of BRCA2) and sixteen available C-terminal domain variants that have been classified by the medical genetics “integrated likelihood ratio” method [1, 8]. We removed the variant R2659K from the validation set because it has been shown to cause defective pre-mRNA splicing [35] and here we are evaluating methods based on ability to predict the functional impact of a variant only on the protein level (Discussion).

### Method comparison

We compared the sensitivity and the specificity of the protein likelihood ratios [30, 31] with four other computational biology methods: SIFT (http://blocks.fhcrc.org/sift/SIFT.html), POLYPHEN (http://genetics.bwh.harvard.edu/pph/), AGVGD (http://agvgd.iarc.fr/), and PMUT (http://mmb2.pcb.ub.es:8080/PMut/) using default parameters [9, 32–34]. To enable direct comparison of methods, we have reduced the multiple classes of Polyphen (“Probably Damaging”, “Possibly Damaging”, “Benign”) and AGVGD (“Enriched Deleterious 1”, “Enriched Deleterious 2”, “Enriched Neutral 1”, “Enriched Neutral 2”) to “Deleterious” or “Neutral.”

## Results

Out of the 229 variants in the C-terminal domains of BRCA2, 127 have protein likelihood ratios below 1.0 (favors neutral) and 102 have protein likelihood ratios above 1.0 (favors deleterious) (Fig. 3a, 3b, Supplementary Table 1). Literature references, population frequencies, and results of a functional assay are available for 22 of the 229 variants (Supplementary Tables 1 and 2). The range of protein likelihood ratios is 0.032 to 202. A likelihood ratio close to 1.0 implies low prediction confidence, because the probabilities of being deleterious and neutral are close to equal. To estimate how far a ratio must be from 1.0 to confidently predict whether deleterious or neutral classifications are favored, we set thresholds based on Myriad Genetics reclassification data. Protein likelihood ratios above ~6.8 or below ~0.6 signify confident predictions. Predictions between these values are considered uncertain. This approach yields 70 predicted deleterious variants, 49 predicted neutral, and 60 with insufficient confidence to predict (Fig. 3a, 3b, Supplementary Table 1). Importantly, the thresholds chosen are based on current data and can be adjusted as new information becomes available.

Based on our current thresholds, 20 of the 26 vari ants in a validation set were confidently predicted as either neutral or deleterious by the Protein Likelihood Ratios. Of these 20 predictions, 18 are in agreement with the validation set. The sensitivity of the Protein Likelihood Ratio was 100% and the specificity was 87% (N = 20) (Table 2). The exact 95% binomial confidence interval around the sensitivity is (48%, 100%). To estimate this sensitivity within a ± 5% range would require a sample of 72 validated deleterious variants. The exact 95% binomial confidence interval around the specificity is (60%, 98%), with a sample of 239 validated neutrals required for specificity within ± 5%. The coverage of the protein likelihood ratios is 77% of the validation set. The six variants that were not classified have likelihood ratios that lie between the current threshold values for confident deleterious and neutral predictions. As more validation data becomes available, we expect that the threshold values for confident predictions will narrow.

Although our coverage is lower than that of the four other computational biology methods evaluated here, we believe it is an advantage that our method is able to identify low confidence predictions and thus avoid making possibly incorrect calls. Overall, Protein Likelihood Ratios and medical genetics methods disagree on only two of the variants in the validation set (Table 2). Four other computational biology methods tested on the validation set appear to be overcalling the number of deleterious variants (Table 2). While all methods have 100% “sensitivity” (agreement with medical genetic methods on five deleterious variants), there is considerable variation between the “specificity” of our method (87%) and that of the other four methods (AGVGD = 52%, SIFT = 50%, PMUT = 46%, POLYPHEN = 36%). However, due to the small sample size of the validation set, two-sided exact binomial 95% confidence intervals on all these statistics are wide, ranging from ± 19% to ± 26%. Statistical certainty ( ± 5% confidence estimate) about Protein Likelihood Ratio agreement with medical genetics would require a sample of 239 validated deleterious variants. For the other methods, it would require a sample of 404 validated deleterious variants (Table 2). Given the limited amount of BRCA2 variants with sufficient genetic evidence for a trusted classification, we are not likely to see these sample sizes in the foreseeable future.

A structural map of all 229 variants in this study, based on our protein homology model, yields an overview of regions of the BRCA2 C-terminal domains that are most sensitive to amino acid changes (Fig. 2). The variants with the highest protein likelihood ratios are enriched in the first oligonucleotide-oligosaccharide-binding (OB1) domain where BRCA2 interacts with the small acid protein DSS1. DSS1 is thought to be critical for the double-stranded DNA repair function of BRCA2 [21, 22, 36]. Importantly, DSS1 is disordered prior to binding to BRCA2 and the stability of BRCA2 requires DSS1 binding [37]. Our classifier was trained on extreme phenotype missense mutants in TP53, which we believe to represent amino acid substitutions and associated local structure environments that destabilize protein structure. No information about the BRCA2-DSS1 interaction was available to our classifier, thus the predicted increased sensitivity to mutation in this region is based only on similarity between general features that impact protein stability seen in 1) deleterious and neutral mutations in our training set (Methods) and 2) variants in the BRCA2 C-terminal domains, with respect to the predictive features used by our classifier. Identification of sensitive regions from a three-dimensional perspective can be helpful in setting a prior probability on the cancer risk associated with variants, based on their structural location. To our knowledge, this study presents the first supporting evidence that homology models, rather than X-ray crystal structures [19, 38–40], can be used to analyze variants in cancer susceptibility genes.

## Discussion

We have presented a new computational approach for analyzing the impact of missense changes in the DNA-binding domains of the cancer susceptibility protein BRCA2 that uses information from protein sequence, structure, and sequence conservation. The raw output of a support vector machine classifier is transformed into a likelihood ratio that can be readily used in a clinical setting and can be combined with likelihood ratios from epidemiology, sequencing and tumor pathology studies to produce an overall odds of causality for a VUS of interest [8]. Although we do not have a gold standard to evaluate our predictions, the approach yielded substantial agreement with classifications from Myriad Genetics and with classifications from a medical genetics integrated likelihood ratio model [1]. The agreement of these independent information sources on a VUS of interest strengthens the inference about its associated cancer risk.

Previous work using structure to predict the impact of VUS in cancer susceptibility genes has relied on the availability of protein X-ray crystal structures [19, 38–40]. Here we show that homology models are useful in this setting, a result that significantly increases the number of genes open to structure-based, bioinformatics VUS analysis, including MLH1, MSH2, (hereditary non-polyposis colorectal cancer) ELAC2 (prostate cancer), PALB2 (breast cancer), and NBS1 (breast and prostate cancer).

Bioinformatics methods can provide fast classifications that do not require pedigree collection, tissue samples, or functional assays. However, we do not expect these methods to be as accurate as results based on medical genetics for an individual VUS of interest. Our results are in general agreement with results of Myriad reclassifications, functional assays, and previously published studies, with a few exceptions (Fig. 3a, 3b, Supplementary Table 1). V2728I, in the likelihood ratio range we have identified as “deleterious”, has been reclassified as a Polymorphism by Myriad Genetics. R2973C, also in our deleterious likelihood range was shown to be competent at homology directed repair in an *in vitro* assay [41] and is predicted neutral in the integrated likelihood model [1]. S2670L, which has one of the highest likelihood ratios in our study has impaired homology directed repair *in vitro* [41], but a histopathology study found loss of heterozygosity (involving loss of the allele carrying the VUS) in breast tumor tissue of a individual with this VUS, a result that has been associated with increased probability of neutrality [38]. We are also in disagreement with the integrated likelihood model for: 1) Y3092C (we classify it as deleterious and they classify it as neutral); 2) R2502C (we classify as neutral and they report 21:1 odds in favor of cancer causality) [1].

A limitation of protein likelihood ratios, and the other computational biology methods referenced in this study, is that we only consider the impact of a change in DNA sequence on protein. These changes may also impact mRNA processing to produce aberrant splice variants and other effects that are not yet understood. To our knowledge, computational tools are not yet able to confidently predict such changes, but efforts in this direction are of great interest to us.

Future directions for our group include studying more VUS in BRCA2 and in other cancer-susceptibility genes. We are assessing whether we can extend our methods to regions of these genes where we lack information about the protein’s three-dimensional shape, using properties of predicted local structure in conjunction with amino acid residue physiochemistry and the evolutionary history of mutated sites.

**Figure 4:**
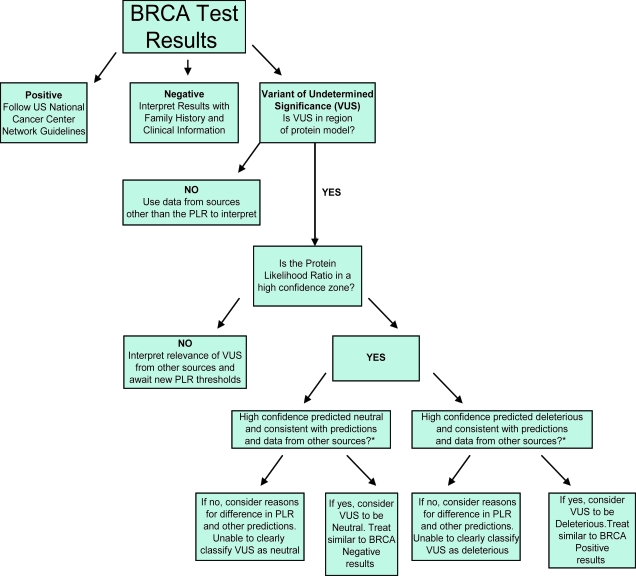
**Flowchart suggesting clinical use for Protein Likelihood Ratio (PLR).** “Variant of Undetermined Significance” (VUS) is one of 3 possible results in BRCA genetic testing [43]. The PLR, when it is a high confidence prediction, and when it agrees with other data predicting whether a VUS is deleterious or neutral, can classify VUS as neutral or deleterious. Neutral VUS can be treated as if BRCA testing was negative, and deleterious VUS can be treated as if BRCA testing was positive. Other data potentially available to compare with VUS predictions include: whether it tracks with cancer in tested family members, whether it has been seen with a known deleterious mutation, whether the tumor shows loss of heterozygosity of the wild-type BRCA2 allele, and predictions from cellular functional studies [1, 8, 38].

Because VUS genetic test results are some of the most difficult to understand for both the provider and the individual, this work represents a first step towards the ability to reclassify VUS in “real time.” Currently, many individuals wait years or longer to learn whether their particular VUS was likely neutral or deleterious. Because individuals use genetic test results to make clinical decisions in “real time,” many individuals are unable to fully utilize genetic test results showing variants of undetermined significance. In Figure 4, we depict a potential flow chart to use clinically, in “real time,” with VUS results. For high confidence predictions that agree with predictions from other methods of VUS reclassification (such as whether it tracks with cancer in tested family members, whether it has been seen with a known deleterious mutation, whether the tumor shows loss of heterozygosity of the wild-type BRCA2 allele, and predictions from cellular functional studies [1, 8, 38]), the protein likelihood ratio (PLR) can aid clinical decision making. VUS predicted deleterious by the PLR, which show consistency with predictions from other VUS reclassification methods can be treated as if the individual tested positive for a BRCA2 deleterious mutation. VUS predicted neutral by the PLR, which show consistency with predictions from other VUS reclassification methods, can be treated as if the individual tested negative for a BRCA2 deleterious mutation. Perhaps the most important issue for future work in this area is rigorous analysis of how to combine and weight predictions from different methods in medical decision making.

In the future, we hope that this research will contribute to quick and accurate classification of genetic results, as a component of predictive algorithms that also include medical genetics information and functional tests, hence bypassing the need to ever have anything labeled as a VUS. Using bioinformatics approaches in the rapidly growing genetic testing arena will require multidisciplinary teams and investigators who can bridge the gap between computational medicine and clinical medicine.

## Supplementary Materials



## Figures and Tables

**Table 1:** **Validation set of variants classified by Myriad Genetics and the medical genetics method of the “integrated likelihood ratio”[1]**is used to compare four computational biology missense variant function prediction methods with the Protein Likelihood Ratio. Incorrect predictions are colored in red. D = Deleterious, N = Neutral, X = insufficient confidence to predict, Myriad = Myriad Genetics (Salt Lake City, Utah), ILR = Integrated Likelihood Ratio, PLR = Protein Likelihood Ratio. SIFT (http://blocks.fhcrc.org/sift/SIFT.html), POLYPHEN (http://genetics. bwh.harvard.edu/pph/), PMUT (http://mmb2.pcb.ub.es:8080/PMut/), AGVGD (http://agvgd.iarc.fr/).

				Predicted classes from four computational biology methods			

Variant	Class	Validation source	PLR	SIFT	POLYPHEN	PMUT	AGVGD
I2627F	D	ILR	D	D	D	D	D
E2663K	D	ILR	D	D	D	D	D
D2665G	N	ILR	N	D	D	D	D
D2723G	D	ILR	D	D	D	D	D
K2729N	N	ILR	X	N	D	D	D
G2748D	D	ILR	D	D	D	D	D
R2842H	N	ILR	X	D	D	D	D
R2888C	N	ILR	N	N	N	D	N
V2908G	N	ILR	X	D	D	D	D
K2950N	N	ILR	N	D	N	N	D
R2973C	N	ILR	D	D	D	D	X
R3052Q	N	ILR	X	D	D	D	D
V3079I	N	ILR	N	N	N	N	N
Y3092C	N	ILR	D	D	D	D	D
Y3098H	N	ILR/Myriad	X	N	N	N	N
D3170G	N	ILR	X	N	N	D	N
I2490T	N	Myriad	N	D	D	N	D
T2515I	N	Myriad	N	D	D	D	N
D2665G	N	Myriad	N	D	D	D	D
A2717S	N	Myriad	N	N	N	N	N
D2723H	D	Myriad	D	D	D	D	D
V2728I	N	Myriad	N	N	N	N	N
S2835P	N	Myriad	N	N	N	N	N
E2856A	N	Myriad	N	N	D	D	N
I2944F	N	Myriad	N	D	D	D	D
T3013I	N	Myriad	N	N	N	D	N

**Table 2:** **Comparison of the Protein Likelihood Ratio with four computational biology methods designed to predict the functional impact of missense variants.**Estimates of sample size required to get sensitivity and specificity within precision of ± 5%, ± 10%, and ± 20% are computed with exact, two-sided binomial confidence intervals. NTP = Number of true positives (correctly predicted Deleterious), NFN = Number of false negatives (Incorrectly predicted Deleterious), NTN = Number of true negatives (correctly predicted Neutrals), NFP = Number of false positives (Incorrectly predicted Neutrals), Sen = Sensitivity NTP/(NTP + NFN), Spec = Specificity NTN/(NTN + NFP), CI = Confidence interval (95% two-sided, exact binomial), Coverage = percent of validation set with confident predictions, N = sample size.

								N	# deleterious for Sen accuracy within ±					N	# neutrals for Spec accuracy within ±		

	NTP	NFN	NTN	NFP	Coverage [%]	Sen [%]	Sen 95% CI*	(deleterious)	5%	10%	20%	Spec [%]	Spec 95% CI*	(neu tral)	5%	10%	20%
PLR	5	0	13	2	77	100	(0.4782,1.000)	5	72	36	17	87	(0.5954,0.9834)	15	239	74	26
AGVGD	5	0	11	10	96	100	(0.4782,1.000)	5	72	36	17	52	(0.2978,0.7429)	21	404	105	27
SIFT	5	0	11	11	100	100	(0.4782,1.000)	5	72	36	17	50	(0.2822,0.7178)	22	402	104	27
PMUT	5	0	10	12	100	100	(0.4782,1.000)	5	72	36	17	45	(0.2439,0.6779)	22	404	106	28
POLYPHEN	5	0	8	14	100	100	(0.4782,1.000)	5	72	36	17	36	(0.1720,0.5934)	22	389	104	29

## Abbreviations

BIC: breast information core database
LR: likelihood ratio
HDR: homology directed repair
OB domain: oligonucleotide/oligosaccharide binding domain
GFP: green fluorescent protein
GEV: generalized extreme value distribution
SVM: support vector machine
