# Supplementary material for: Classifying Variants of Undetermined Significance in BRCA2 with Protein Likelihood Ratios
Source: Cancer Inform. 2008 Apr 18;6:203–16. doi: 10.4137/cin.s618 (PMC2587343; doi:10.4137/cin.s618)
Supplement: Supplementary file 1 [file cin-6-0203-s1.pdf]

Supplementary Table 1. Protein likelihood ratios for 223 VUS in C-terminal DNA binding domain of BRCA2 from BIC. Protein LR = likelihood ratio in favor of protein loss of function. †Seen in one individual in a healthy control population of 180 (Australian Breast Cancer Family Study). **HDR = homology directed repair assay [1] (Supplementary Table 2) ††LOH (loss of heterozygosity) in tumor tissue of variant allele increases probability that the variant is neutral. XOdds of causality from the Goldgar integrated likelihood method [2]. ***Myriad Genetics classifications provided by Amie Deffenbaugh.

Variants	Protein LR	Protein LR Prediction	Myriad***	Integrated Model Odds of Causality[X]	Functional	References
L2480V	0.034	Neutral				
S2483G	0.038	Neutral				
S2483N	0.035	Neutral				
R2488S	2.705	Uncertain				
R2488K	0.131	Neutral				
D2489G	0.119	Neutral				
I2490T	0.033	Neutral	Polymorphism			High BIC frequency
R2494Q	24.867	Likely deleterious				
K2496T	0.499	Neutral				
R2500T	0.451	Neutral				
R2502C	0.175	Neutral		21		
R2502P	0.099	Neutral				
R2502H	0.095	Neutral				
F2504L	0.038	Neutral				
G2508S	0.273	Neutral				
L2510P	1.497	Uncertain				
L2512F	0.055	Neutral				
T2515I	0.074	Neutral	Polymorphism			High BIC frequency
R2520Q	1.216	Uncertain				
I2521T	0.040	Neutral				
I2521V	0.033	Neutral				
S2522F	23.905	Likely deleterious				
G2528E	0.106	Neutral				
S2533C	1.292	Uncertain				
A2534V	2.446	Uncertain				
H2537R	0.033	Neutral				
T2542R	0.035	Neutral				
V2545I	1.336	Uncertain				
K2547E	0.535	Neutral				
H2548R	0.032	Neutral				
F2562L	0.054	Neutral				
D2566Y	0.074	Neutral				
E2571G	0.086	Neutral				
L2581W	1.269	Uncertain				
G2584D	6.239	Uncertain				
G2585R	4.722	Uncertain				
P2589H	2.873	Uncertain				
N2591S	0.035	Neutral				
A2595S	0.113	Neutral				
T2607P	11.912	Likely deleterious				
G2609D	33.476	Likely deleterious				
D2611G	0.085	Neutral				
H2623R	17.554	Likely deleterious				

Variants	Protein LR	Protein LR Prediction	Myriad***	Integrated Model Odds of Causality^{X}	Functional	References
W2626C	100.037	Likely deleterious		48		
I2627F	17.940	Likely deleterious		1046		
P2639A	1.901	Uncertain				
A2643V	0.101	Neutral				
A2643G	0.070	Neutral				
N2644S	0.057	Neutral				
L2647P	8.778	Likely deleterious				
L2653P	69.681	Likely deleterious		24		
L2654P	9.634	Likely deleterious				
Q2655R	14.769	Likely deleterious				
R2659G	186.215	Likely deleterious				
R2659T	160.838	Likely deleterious	Suspected Deleterious	3339		Predicted as deleterious in [1]
R2659K	4.629	Uncertain				Produces aberrant splicing [3]
Y2660D	49.121	Likely deleterious				
E2663V	70.926	Likely deleterious		233		
E2663K	18.055	Likely deleterious				
D2665G	0.168	Neutral	Polymorphism	0.002		Enriched in a healthy control population† and LOH in variant allele†† [4]
R2666T	0.216	Neutral				
S2670L	191.227	Likely deleterious			impaired HDR**	LOH in variant allele†† [4]
I2672V	0.246	Neutral				
M2676T	0.044	Neutral			competent HDR**	
R2678G	18.113	Likely deleterious				
D2679Y	52.441	Likely deleterious				
D2679G	41.704	Likely deleterious				
D2680G	0.096	Neutral				
K2684R	0.145	Neutral				
L2686P	42.616	Likely deleterious				
V2687F	170.909	Likely deleterious				
L2688P	191.184	Likely deleterious				
S2691F	25.395	Likely deleterious				
S2695L	1.007	Uncertain				
S2697N	0.043	Neutral				
I2700L	0.032	Neutral				
S2704F	0.053	Neutral				
N2706S	0.048	Neutral				
D2712N	0.056	Neutral				
D2712V	0.043	Neutral				
Q2714R	0.038	Neutral				
A2717S	0.045	Neutral	Polymorphism			High BIC frequency
I2718T	0.251	Neutral				
L2721H	98.117	Likely deleterious			impaired HDR**	

Variants	Protein LR	Protein LR Prediction	Myriad***	Integrated Model Odds of Causality^X	Functional	References
T2722R	35.193	Likely deleterious		93		
D2723G	65.119	Likely deleterious				
D2723A	55.652	Likely deleterious		121	impaired HDR**	
D2723H	13.116	Likely deleterious	Deleterious		impaired HDR**	Predicted as likely deleterous in [2]
G2724V	26.336	Likely deleterious				
Y2726C	31.697	Likely deleterious				
V2728I	0.615	Neutral	Polymorphism			High BIC frequency
V2728L	0.123	Neutral				
K2729N	3.040	Uncertain		0.002		
A2730P	178.756	Likely deleterious				
A2730V	28.610	Likely deleterious				
V2739I	0.033	Neutral			competent HDR**	
G2748D	6.830	Likely deleterious		2494		
I2752F	0.231	Neutral				
M2775R	0.120	Neutral				
M2775T	0.047	Neutral				
R2784W	118.033	Likely deleterious				
R2784Q	7.243	Likely deleterious				
A2786P	95.379	Likely deleterious				
R2787C	115.727	Likely deleterious				
R2787H	0.342	Neutral				
W2788S	45.999	Likely deleterious				
W2788R	21.840	Likely deleterious				
T2790S	1.870	Uncertain				
L2792P	201.610	Likely deleterious				
G2793E	9.375	Likely deleterious				
G2793R	7.648	Likely deleterious				
F2794L	0.116	Neutral				
P2800R	0.501	Neutral				
P2800S	0.188	Neutral				
S2807L	0.996	Uncertain				
S2810G	0.042	Neutral				
D2811G	0.033	Neutral				
G2812E	7.139	Likely deleterious				
G2813E	79.102	Likely deleterious				
V2815I	0.617	Uncertain				
V2818I	0.103	Neutral				
V2820L	26.066	Likely deleterious				
I2821T	0.978	Uncertain				
I2828V	0.050	Neutral				
Q2829L	0.074	Neutral				
S2835P	0.033	Neutral	Polymorphism		competent HDR**	

Variants	Protein LR	Protein LR Prediction	Myriad***	Integrated Model Odds of Causality^{X}	Functional	References
I2840V	0.035	Neutral				
R2842C	184.961	Likely deleterious				
R2842L	39.694	Likely deleterious		6.35E-04		
R2842H	1.148	Uncertain				
N2843S	0.103	Neutral				
E2847K	3.239	Uncertain				
K2849E	1.854	Uncertain				
E2850K	0.383	Neutral				
E2856A	0.073	Neutral	Polymorphism			Enriched in a healthy control population† and LOH in variant allele†† [4]. High BIC frequency.
Q2858K	0.660	Uncertain				
Q2858R	0.036	Neutral				
A2864V	0.128	Neutral				
L2865V	0.230	Neutral				
T2867P	0.397	Neutral				
F2873C	0.977	Uncertain				
P2883S	0.032	Neutral				
R2888L	1.112	Uncertain		8.00E-04		
R2888C	0.447	Neutral				
R2888P	0.311	Neutral				
A2889S	0.040	Neutral				
L2890I	0.073	Neutral				
R2896H	0.086	Neutral			competent HDR**	
G2901D	5.954	Uncertain				
V2908G	1.879	Uncertain		0.004	competent HDR**	
A2911V	0.141	Neutral				
G2919V	0.034	Neutral				
S2922G	0.743	Uncertain				
Q2925R	6.931	Likely deleterious				
Q2925H	5.282	Uncertain				
L2936M	0.111	Neutral				
L2936F	0.071	Neutral				
A2942T	0.087	Neutral				
I2944F	0.199	Neutral	Polymorphism			High BIC frequency
K2950N	0.537	Neutral		7.15E-10		High BIC frequency
A2951T	0.074	Neutral				
D2965H	0.091	Neutral				
V2969M	17.047	Likely deleterious				
L2972W	107.825	Likely deleterious				
R2973C	70.762	Likely deleterious		1.75E-04	competent HDR**	
R2973H	0.324	Neutral				
K2982Q	0.077	Neutral				
S2988G	14.558	Likely deleterious				
R2991H	0.433	Neutral				
E3002D	84.493	Likely deleterious				
E3002K	33.891	Likely deleterious				
L3011P	118.966	Likely deleterious				
T3013I	0.247	Neutral	Polymorphism			High BIC frequency
S3020C	0.042	Neutral				
Q3026E	0.727	Uncertain				
A3028P	0.034	Neutral				

Variants	Protein LR	Protein LR Prediction	Myriad***	Integrated Model Odds of Causality^X	Functional	References
A3029V	0.246	Neutral				
A3029T	0.133	Neutral				
Q3034R	0.035	Neutral				
Y3035S	165.485	Likely deleterious				
Y3035C	164.959	Likely deleterious				
P3039L	0.074	Neutral				
V3040I	0.320	Neutral				
R3052W	33.692	Likely deleterious		0.003		
R3052Q	2.166	Uncertain				
P3054H	1.734	Uncertain				
S3058G	0.048	Neutral				
K3059E	3.937	Uncertain				
P3063S	0.035	Neutral				
D3064Y	0.062	Neutral				
D3064N	0.036	Neutral				
F3065L	0.032	Neutral				
Q3066E	0.059	Neutral				
E3071D	1.664	Uncertain				
V3072E	18.936	Likely deleterious				
D3073G	12.265	Likely deleterious				
G3076E	12.781	Likely deleterious				
G3076V	5.000	Uncertain			impaired HDR**	
V3079I	0.069	Neutral		6.47E-04		
V3081A	0.400	Neutral				
K3083E	0.235	Neutral				
K3083N	0.072	Neutral				
A3088V	7.760	Likely deleterious				
V3091S	9.047	Likely deleterious				
Y3092C	110.954	Likely deleterious		0.006		
Y3092S	22.321	Likely deleterious				
D3095E	11.009	Likely deleterious		23		
Y3098H	1.041	Uncertain	Favor Polymorphism	3.46E-04		
L3101R	36.547	Likely deleterious				
L3101V	0.119	Neutral				
I3103M	0.299	Neutral				
I3103V	0.053	Neutral				
H3117P	4.212	Uncertain				
M3118T	0.034	Neutral				
S3123G	5.272	Uncertain				
N3124I	83.538	Likely deleterious				
L3125H	111.532	Likely deleterious				
P3129A	0.043	Neutral				
G3134V	0.302	Neutral				
D3142G	0.059	Neutral				
P3150L	5.063	Uncertain				
E3152G	0.694	Neutral				
E3152K	0.084	Neutral				
G3153A	0.035	Neutral				
F3159L	0.051	Neutral				

Variants	Protein LR	Protein LR Prediction	Myriad***	Integrated Model Odds of Causality^X	Functional	References
E3167A	0.069	Neutral				
D3170G	2.060	Uncertain		0.001		
M3181R	0.228	Neutral				
D3188N	0.034	Neutral				

References

1. Wu, K., et al., *Functional evaluation and cancer risk assessment of BRCA2 unclassified variants.* Cancer Res, 2005. **65**(2): p. 417-426.
2. Easton, D.F., et al., *A Systematic Genetic Assessment of 1,433 Sequence Variants of Unknown Clinical Significance in the BRCA1 and BRCA2 Breast Cancer Predisposition Genes.* American Journal of Human Genetics, 2007. **Early Access**.
3. Hofmann, W., et al., *The BRCA2 variant 8204G>A is a splicing mutation and results in an in frame deletion of the gene.* J Med Genet, 2003. **40**(3): p. e23.
4. Chenevix-Trench, G., et al., *Genetic and Histopathologic Evaluation of BRCA1 and BRCA2 DNA Sequence Variants of Unknown Clinical Significance.* Cancer Research, 2006. **66**(4): p. 2019-2027.

Supplementary Table 2. Homology-directed repair assay results for eleven BRCA2 variants using direct repeat (DR) green fluorescent protein (GFP). wt = wild-type. Fold increase in GFP+ cells counted by Fluorescent-Activated Cell Sorting (FACS) 72 hours after transfection of VC8-DR-GRP cells. 3X = three repetitions of the experiment [1]. Impaired homologous recombination is defined as fold increase in GFP positive cells after 72 hours < 2.0. and competent homologous recombination as fold increase > 3.5.

	Fold increase in number of GFP+ cells	Standard error (3X)
Vector	1.0	0.1
BRCA2-wt	5.4	0.2
S2670L	1.9	0.1
M2676T	3.9	0.6
L2721H	1.7	0.1
D2723G	1.3	0.1
D2723H	1.4	0.1
K2729N	4.4	0.5
V2739I	3.8	0.2
R2896H	5.2	0.7
V2908G	5.3	0.1
R2973C	4.2	0.4
G3076V	1.8	0.1

References

1. Wu, K., et al., *Functional evaluation and cancer risk assessment of BRCA2 unclassified variants.* Cancer Res, 2005. **65**(2): p. 417-426.
